# Macrophage and Neutrophil Interactions in the Pancreatic Tumor Microenvironment Drive the Pathogenesis of Pancreatic Cancer

**DOI:** 10.3390/cancers14010194

**Published:** 2021-12-31

**Authors:** Hillary G. Pratt, Kayla J. Steinberger, Nicole E. Mihalik, Sascha Ott, Thomas Whalley, Barbara Szomolay, Brian A. Boone, Timothy D. Eubank

**Affiliations:** 1 Cancer Cell Biology, West Virginia University, Morgantown, WV 26506, USA; hp0021@mix.wvu.edu; 2 WVU Cancer Institute, West Virginia University, Morgantown, WV 26506, USA; 3 Department of Microbiology, Immunology and Cell Biology, West Virginia University, Morgantown, WV 26506, USA; kjp0007@mix.wvu.edu (K.J.S.); nem0007@mix.wvu.edu (N.E.M.); 4 Warwick Medical School, University of Warwick, Coventry CV4 7AL, UK; s.ott@warwick.ac.uk; 5 School of Biosciences, Cardiff University, Cardiff CF14 4XN, UK; whalleyt@cardiff.ac.uk; 6 Division of Infection and Immunity & Systems Immunity Research Institute, Cardiff University, Cardiff CF14 4XN, UK; SzomolayB@cardiff.ac.uk; 7 Department of Surgery, West Virginia University, Morgantown, WV 26506, USA; 8 The In Vivo Multifunctional Magnetic Resonance Center, West Virginia University, Morgantown, WV 26506, USA

**Keywords:** macrophage, neutrophil, immunosuppression, hypoxia, metastasis, cancer, adenocarcinoma, PDAC, pancreas

## Abstract

**Simple Summary:**

The survival rates for patients with pancreatic adenocarcinoma are very low. This dismal prognosis is due in part to late detection and early development of metastases, and successful treatments for pancreatic adenocarcinoma are also lacking. One potential method of treatment is immunotherapy, which has been successfully implemented in several cancers. Despite success in other cancer types, there has been little progress in pancreatic adenocarcinoma. To understand these shortcomings, we explore the roles of macrophages and neutrophils, two prominent immune cell types in the pancreatic tumor environment. In this review, we discuss how macrophages and neutrophils lead to the harsh environment that is unique to pancreatic adenocarcinoma. We further explore how these immune cells can impact standard of care therapies and decrease their effectiveness. Macrophages and neutrophils could ultimately be targeted to improve outcomes for patients with pancreatic adenocarcinoma.

**Abstract:**

Despite modest improvements in survival in recent years, pancreatic adenocarcinoma remains a deadly disease with a 5-year survival rate of only 9%. These poor outcomes are driven by failure of early detection, treatment resistance, and propensity for early metastatic spread. Uncovering innovative therapeutic modalities to target the resistance mechanisms that make pancreatic cancer largely incurable are urgently needed. In this review, we discuss the immune composition of pancreatic tumors, including the counterintuitive fact that there is a significant inflammatory immune infiltrate in pancreatic cancer yet anti-tumor mechanisms are subverted and immune behaviors are suppressed. Here, we emphasize how immune cell interactions generate tumor progression and treatment resistance. We narrow in on tumor macrophage (TAM) spatial arrangement, polarity/function, recruitment, and origin to introduce a concept where interactions with tumor neutrophils (TAN) perpetuate the microenvironment. The sequelae of macrophage and neutrophil activities contributes to tumor remodeling, fibrosis, hypoxia, and progression. We also discuss immune mechanisms driving resistance to standard of care modalities. Finally, we describe a cadre of treatment targets, including those intended to overcome TAM and TAN recruitment and function, to circumvent barriers presented by immune infiltration in pancreatic adenocarcinoma.

## 1. Introduction

The 5-year survival rate for patients with pancreatic ductal adenocarcinoma (PDAC) is only 9%. The incidence of PDAC is increasing, and it is projected to become the second leading cause of cancer deaths by 2030 [1]. Meanwhile, the only known curative treatment for PDAC is surgical resection, but only 20% of patients are surgical candidates at the time of presentation [2]. Given the rising incidence of PDAC and currently limited therapeutic success, additional strategies for combatting this deadly disease are desperately needed. Unique to PDAC, the tumor microenvironment (TME) drives therapeutic resistance through its highly fibrotic and immunosuppressive nature. In order to improve therapeutic response in PDAC, it is important to understand immune infiltrate function in the pathophysiology of PDAC.

## 2. Significance of the Inflammatory Immune Infiltrate in PDAC

The PDAC TME is marked by a fibrotic stroma which contains an array of immune cells. Many studies have explored the immune cell infiltrate and how its composition affects PDAC patient outcomes and survival. Importantly, many of the immune cells within the TME are immunosuppressive while immunosupportive cells are either excluded or reprogrammed [3,4,5,6,7,8,9,10]. T cells in particular have been widely studied regarding their role in PDAC progression. Regulatory T cells (Tregs) are known for their immunosuppressive qualities, and high staining for FoxP3^+^ Tregs correlates with advanced tumor stage, distant metastases, and decreased overall survival [5,7]. In general, higher CD4^+^ and CD8^+^ infiltrating T cells predict a better prognosis and improved overall survival [5,6]. Although anti-tumorigenic CD8^+^ T cells can be found in resected PDAC specimens, they are commonly located in the fibrotic interstitial regions of the tumor [8], isolated from the cancer cells. This is further demonstrated by the low efficacy of immune checkpoint blockade treatments in patients with PDAC. While expression of immune checkpoint molecule cytotoxic lymphocyte associated antigen 4 (CTLA-4) on CD8^+^ T cells is associated with a shorter overall survival [11], dual checkpoint blockade therapy of CTLA-4 and programmed death-ligand 1 (PD-L1) did not have significant efficacy in patients with metastatic PDAC [12]. Thus, further studies of CD8^+^ T cell interactions are warranted in PDAC. For example, CD8^+^ T cells correlate with the higher density of CD20^+^ B cells organized in tertiary lymphoid structures within PDAC. Importantly, these B cell clusters in the PDAC TME correlate with better overall survival. On the other hand, however, the presence of B cells scattered throughout the tumor correlates with a worse prognosis. In the B cell tertiary lymphoid structures within in the PDAC TME, both T cells and mature dendritic cells can be found, suggesting an important immunologic function of these structures [13]. Overall, these studies demonstrate that the adaptive immune system plays a prominent role in PDAC, although the function of these cells may be suppressed by other components of the TME. 

Cells of the innate immune system are also present in the PDAC TME and play a role in patient outcomes. Dendritic cells, known for their ability to activate T cells, have been identified as an independent prognostic factor for patients with PDAC. Patients with higher circulating myeloid dendritic cells have a longer overall survival [14]. Further, patients at stages 3 and 4 of disease have lower activity of myeloid dendritic cells compared to patients at earlier stages of disease [15]. Mast cells can also be used to predict outcomes for patients with PDAC. In general, mast cells have pro-tumorigenic properties in the TME [16]. For pancreatic cancer, increased mast cell staining in the tumor is associated with a worse prognosis [17,18]. Myeloid-derived suppressor cells (MDSCs), immunosuppressive specifically toward T cells, also appear to play an important role in PDAC prognosis and outcomes. Monocytic (M)-MDSCs and polymorphonuclear (PMN)-MDSCs related to macrophage lineage cells and neutrophil lineage cells, respectively, are higher in circulation of patients with PDAC compared to healthy patients. These cells also infiltrate the PDAC TME [19]. Specifically, when CD11b^+^CD15^+^ PMN-MDSCs infiltration is elevated, CD4^+^ and CD8^+^ T cell infiltration is decreased in PDAC [9]. Macrophages and neutrophils are also innate immune cells found to be critical to the pathogenesis of pancreatic cancer. In a meta-analysis of gene expression across 39 different malignancies, including PDAC, infiltrating granulocytes were found to be the most significant predictor of an adverse prognosis. M2-like tumor-associated macrophages (TAMs) also predict adverse outcomes [20]. Given the importance of macrophages and neutrophils, we sought to identify how these macrophages and neutrophils, independently and through their interactions, influence the PDAC TME.

**Figure 1:**
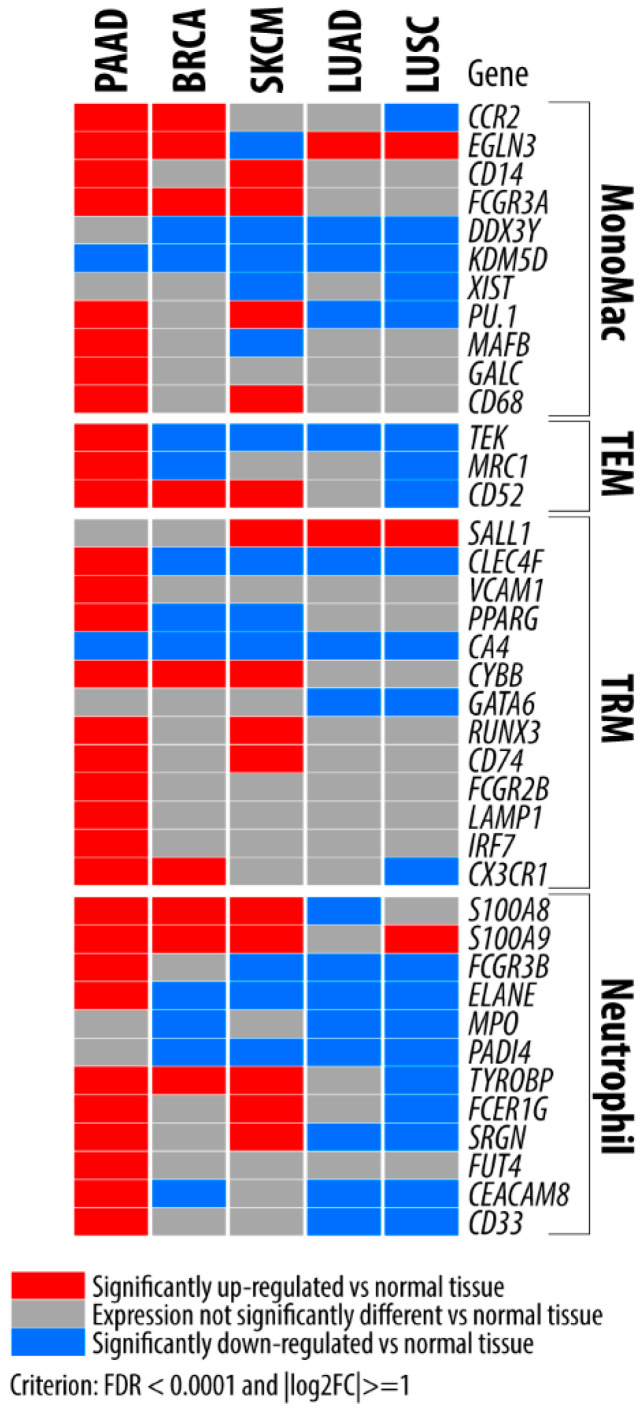
Gene expression in tumor type relative to its normal tissue. We used the TCGA TARGET GTEx cohort from the UCSC Xena platform [21], which has 60,498 gene variants in total and combines normalized samples from both TCGA and GTEx. Since the data in [22] has log_2_(x + 1)-transformed RSEM expected count values, the exponent was taken, and a unity subtracted to obtain the isoform-level estimates. Five TCGA projects (PAAD, pancreatic adenocarcinoma; BRCA, breast invasive carcinoma; SKCM, skin cutaneous melanoma; LUAD, lung adenocarcinoma; LUSC, lung squamous cell carcinoma) containing primary tumor samples were compared using [27]. By taking the overlap of TCGA primary tumor sample IDs and the TCGA TARGET GTEx sample IDs, we obtained PAAD (*n* = 178), BRCA (*n* = 1092), SKCM (*n* = 102), LUAD (*n* = 512), LUSC (*n* = 498) samples for each. From the TCGA TARGET GTEx cohort four non-disease tissue sites were extracted: pancreas (*n* = 155), breast (*n* = 165), skin (*n* = 501), lung (*n* = 247) by searching for the relevant tissue in the “SMTSD” column and RNASEQ keyword in the “SMAFRZE” column of the sample annotation file from the GTEx Portal [26] and then taking the overlap with the TCGA TARGET GTEx sample IDs. The differential expression analysis compared the TCGA data to the GTEx data and was performed using edgeR [23,25]. We define significant genes from the 39 genes of interest if the false discovery rate (FDR) is less than 0.0001 and the log2-fold change is at least ±1. Most significant genes, 33 in total, were identified for the PAAD cohort and less than 26 significant genes for each of the BRCA, SKCM, LUAD, and LUSC projects. *MonoMac,* bone marrow-recruited monocytes differentiating to macrophages; *TEM*, TIE2-expressing macrophages; *TRM*, a priori tissue resident macrophages.

To begin understanding the importance of macrophages and neutrophils in the PDAC TME, we analyzed publicly available datasets from the University of California Santa Cruz (UCSC) Xena platform [21,22] and found that the gene signatures for recruited monocyte-derived macrophages (MonoMacs), endothelial tyrosine kinase receptor (TIE)2-expressing macrophages (TEMs), and a priori tissue-resident macrophages (TRMs) as well as neutrophils are increased when compared to normal pancreatic tissue (Figure 1) [21,22,23,24,25,26]. This increase is unique to PDAC compared to other cancer types and warrants further investigation to enhance our understanding of how these cells interact and potentially uncover new therapeutic targets. We hypothesize that the chronic, unresolving inflammation driven by macrophages and neutrophils, as well as possible atypical interactions between these two cell types, is unique to PDAC and leads to the fibrosis and immunosuppression seen in the TME. In this review, we identify the roles of MonoMacs, TRMs, TEMs, and neutrophils in PDAC carcinogenesis and potential sequelae of their interactions in the PDAC TME.

## 3. Importance of Murine Models in Studying the PDAC Tumor Microenvironment 

To study the tumor immune infiltrate in PDAC, it is important to recapitulate the PDAC TME in pre-clinical murine models. The PDAC TME is characterized by a dense, hypoxic and fibrotic stroma that can make up 90% of the tumor mass [28]. Since the TME plays a substantial role in therapeutic resistance in pancreatic cancer, the ability of murine models to reliably mimic the heterogenous nature of PDAC has become increasingly important [29,30,31,32,33,34,35]. Development of PDAC genetically engineered mouse models (GEMMs) has been important for recapitulating the heterogenous nature of PDAC in murine models. Common mutations in human pancreatic cancer, including the proto-oncogene *K-ras* and the tumor suppressor genes *CDKN2A*, *p53*, and *DPC4/SMAD4*, can be exploited for development of PDAC GEMMs. In one murine model of pancreatic cancer, commonly referred to as the KC model, pancreatic/duodenal homeobox protein-1 (PDX-1)-expressing pancreatic progenitor cells harbor a *K-ras* mutation (*Pdx-Cre*; *K-ras^LSL^*^.G12D/+^). These mice are born with normal pancreatic histology but develop PanIN lesions by 8 weeks. These PanIN lesions are histologically similar to those seen in human pancreatic cancer progression. Importantly, the PanIN lesions slowly progress to pancreatic cancer over a period of 2 years. In this model, the fibrotic stroma is similar to that seen in human PDAC, and metastases develop in the liver, lung, and diaphragm. Although this model recapitulates the disease progression of PDAC, heterogenous disease progression has been noted in these animals and some animals did occasionally develop tumors in other areas, such as mucocutaneous papillomas, intestinal metaplasia of the gastric epithelium, and duodenal polyps, due to PDX-1 expression. Comparatively, use of the PTF1a-p48 (p48) promoter (*P48^+/Cre^*; *K-ras^LSL^*^.G12D/+^), which commits pancreatic progenitor cells at a later stage in pancreatic development, results in fewer tumors outside of the pancreas. Another important difference between the *Pdx-Cre*; *K-ras^LSL^*^.G12D/+^ and *P48^+/Cre^*; *K-ras^LSL^*^.G12D/+^ models is where the mutation is found throughout the cells of the pancreas. While the *Pdx-Cre*; *K-ras^LSL^*^.G12D/+^ model harbors the *K-ras* mutation at random locations throughout the pancreas, the mutation is harbored uniformly through the pancreas in the *P48^+/Cre^*; *K-ras^LSL^*^.G12D/+^ model [35]. Together, these models demonstrated that a *K-ras* mutation alone is sufficient to cause PanIN lesions with progression to invasive cancer and metastatic PDAC [34,35]. The stepwise progression of pancreatic cancer can also be recapitulated with the KPC murine model (*Pdx-Cre*; *K-ras*^*LSL*.G12D/+^; *p53^R17H/+^*), which targets another common mutation seen in human PDAC, p53. These mice develop metastases in the liver, lung, diaphragm, and peritoneum. However, addition of the p53 mutation in this model accelerates the timeline of cancer progression, and mice develop significant disease by 10 weeks of age and have a median survival time around 5 months. Factors known to be important to pancreatic tumorigenesis were found to be present in the KPC model, including Sonic hedgehog (*Shh*) and tyrosine kinase *Errb2/HER2* gene expression and chromosomal instability. One drawback to this model, however, is the presence of esophageal papillomas and hyperplasia of the biliary tree, which has been attributed to *PDX-1* expression in early development of the foregut [34]. These GEMMs and other models for PDAC have been more thoroughly reviewed elsewhere [31,32,36]. 

In addition to GEMMs, both orthotopic syngeneic and patient derived xenograft (PDX) models have been used [31]. Unlike GEMMs, however, these models fail to adequately model the unique TME found in human PDAC [30,31,32]. For example, gemcitabine therapy decreases tumor volume in both subcutaneous syngeneic and orthotopic PDX models. However, treatment of KPC GEMMs with gemcitabine does not affect tumor volume in the majority of treated mice, emulating what is seen in human PDAC patients. Resistance in the KPC model is driven by the fibrotic stroma, which limits vascularization and thus delivery of gemcitabine into the tumor, rather than some tumor cell intrinsic mechanism. Importantly, the vascularization of human PDAC tumors is most similar to that of KPC model tumors compared to other models [29]. Fibrosis and vascularization can also impact the ability of immune cells to infiltrate the PDAC TME. Indeed, the immune infiltrate in orthotopic and xenograft models does not recapitulate human disease making it challenging to reliably study immunotherapies in these models [30,31,32,37]. For example, B cells infiltrate the PDAC TME of the KPC GEMM at a sixfold higher rate than that of orthotopic injection of KPC-derived tumor cells [30]. Similarly, bone marrow-derived neutrophils isolated from a GEMM model have greater spontaneous migratory capacity than those isolated from a subcutaneous injectable model of KPC.4662 cells [37]. PDX models may more reliably recapitulate the PDAC TME than syngeneic orthotopic models [38]; however, even in humanized mouse model with engrafted CD34^+^ human hematopoietic stem cells, residual murine immune cells can impact the immune cell infiltrate and function [32]. Further, given the potential interactions between immune cells within the PDAC TME, it is important to reliably replicate the TME to understand those spatial interactions. Together, these studies demonstrate the importance of using clinically relevant murine models for studying therapeutics in PDAC. 

## 4. Introduction to Macrophages in PDAC

Macrophages commonly make up a dominant proportion of the immune cell infiltrate in the PDAC TME [39]. Given the prominence of these cells, it is important to understand their ontogeny, phenotype, and function. Such insight will provide valuable information regarding how macrophages affect pancreatic carcinogenesis and can potentially be targeted to improve outcomes for patients with PDAC. 

Macrophage ontogeny, or its source of origin, has been recently explored for playing an important role in phenotype and function. The most commonly discussed source of macrophages in tissues, including the pancreas, is monocytes from the circulation, derived from the bone marrow [10,39]. In this review, we will refer to macrophages derived from the circulation as MonoMacs (Table 1). In general, circulating monocyte subsets are classified prior to infiltrating tissue by their expression intensity of the immune co-receptors CD14 and CD16 [40]. The pattern recognition receptor CD14 acts in complex with the endotoxin lipopolysaccharide (LPS)-binding protein (LBP) as a first line of defense against invading gram-negative pathogens, such as *E. coli*, *Pseudomonas*, and *Legionella*. CD14/LPB/LPS complexation with Toll-like receptor 4 (TLR4) and myeloid differentiating factor 2 (MD2) triggers activation of NF-κB and other classic pro-inflammatory signaling events to produce interleukin (IL)-12 and type 1 interferons [41]. CD16, commonly referred to as Fc-gamma Receptor III (FcγRIII), is a transmembrane receptor on monocytes that induces phagocytosis and oxidative burst. While FcγRIIIa is expressed on monocytes, FcγRIIIb is expressed solely on neutrophils and regulates degranulation to support pathogen clearance [42]. CD14 and CD16 expression delineate the three major monocyte subsets in humans, which are functionally distinct (two monocyte subsets in mice): Elevated CD14 expression and CD16 absence (CD14^++^ CD16^−^ CCR2^hi^) defines classical monocytes that comprise about 80–95% of circulating monocytes in humans and have been reported to be equivalent to murine inflammatory monocytes (CX3CR1^lo^ Ly6C^hi^ CCR2^+^). These classical monocytes also express the monocyte chemokine receptor C-C Motif Chemokine Receptor 2 (CCR2) and migrate in response to C-C Motif Chemokine Ligand 2 (CCL2) produced during episodes of inflammation. Importantly, elevated levels classical/inflammatory monocytes have been correlated with worse prognosis in patients with PDAC as they are recruited into the TME where they become immunosuppressive macrophages, as further discussed below [10]. Elevated CD16 expression (CD14^+^ CD16^++^ CCR2^−^) identifies a “non-classical” monocyte subset that comprise 2–11% of circulating monocytes. These non-classical monocytes replenish specialized macrophages such as alveolar macrophages in the lung or accumulate in tissues under chronic inflammation such as in Crohn’s disease, wound healing, and malignancy [43,44]. Human non-classical monocytes are equivalent to murine tissue resident macrophages (CX3CR1^hi^ Ly6C^lo^ CCR2^−^) [45] to be discussed below. Given the importance of classical monocytes in PDAC prognosis, further studies are warranted into other classes, including “non-classical” monocytes and tissue resident macrophages.

While monocytes make up one source of macrophages (MonoMacs) in tissues, macrophages can reside and replicate within the tissue as a priori tissue resident macrophages (TRMs) as defined in Figure 1/Table 1. Specifically, in the pancreas, TRMs can maintain their population in the pancreatic tissue without repletion by circulating MonoMacs under physiological conditions. Exploiting the Myb transcription factor, which is important for development of hematopoietic stem cells and monocytes, Schulz et al. showed that approximately half of the macrophages in the pancreas are derived from primitive hematopoiesis, and only 10% of all pancreatic macrophages are replaced by circulating MonoMacs under physiological conditions [46]. Macrophages within the islets of Langerhans, on the other hand, are derived from adult hematopoiesis, suggesting that neither stromal macrophages nor islet macrophages commonly undergo turnover and replacement by CCR2^+^ MonoMacs. Healthy CCR2^−/−^ mice, having deficient MonoMac recruitment, have no difference in the population of macrophages within islets and stroma. [47]. These findings demonstrate the importance of understanding macrophage origin in both the healthy pancreas and pancreatic pathologies, such as PDAC. While ontogeny is certainly important for understanding macrophage function and should be explored in future studies, macrophages have historically been classified in terms of their polarization. 

Macrophages can also be characterized by their phenotype or expression of particular cell surface markers. Although tumor macrophage polarization occurs along a continuum, most studies characterize macrophages as M1-like or M2-like [48]. M1-like macrophages are typically indicated by expression of markers such as iNOS, HLA-DR, CD80, and CD86 (Table 1) and promote a Th1, anti-tumorigenic or immunostimulatory response. In contrast, M2-like macrophages are commonly identified by expression of Arginase (ARG)-1, CD163, and CD206 and promote a Th2, pro-tumorigenic or immunosuppressive response [49,50]. M2-like macrophages have immunosuppressive functions and are referred to as TAMs in the PDAC TME (Table 1). Another important subtype of macrophages includes those expressing endothelial receptor TIE2 (TEMs) [51] that are specific for the four angiopoietin ligands (ANG1-4) [52]. TIE2/*TEK* was once thought to be solely expressed on cells comprising the endothelium until the discovery of their expression on a myeloid population, TEMs (Table 1) [53]. Most relevant to this review are ANG1 and ANG2 as their ratio in a hypoxic tumor setting (high ANG2:low ANG1) regulate important differences between physiological angiogenesis and pathological vascular dysfunction sustaining hypoxia that predicts worse prognosis in patients with solid tumors, including breast cancer and pancreatic cancer [54,55,56,57,58,59]. In particular, TIE2 monocytes/macrophages differ from other bone marrow-derived monocytes as they lack CCR2 expression and are not recruited via the CCR2/CCL2 axis. Instead, TEMs are primarily recruited via the TIE2/ANG2 axis and play a role in the formation of new blood vessels from pre-existing vessels, a hallmark of cancer known as angiogenesis [60,61]. It is important to note that classifying macrophages by their phenotypic expression alone is a gross oversimplification of their polarization, but these commonly used paradigms will be used throughout the review to understand the spectrum of macrophage polarization in the PDAC TME as outlined in Table 1.

Macrophages in PDAC are primarily composed of M2-like TAMs and are known for having immunosuppressive or pro-tumorigenic qualities. Increased infiltration of CD163^+^ TAMs in patients with pancreatic cancer is associated with a lower rate for 5-year survival and 5-year recurrence free survival, but the general presence of macrophages, designated by the pan-macrophage marker CD68, does not correlate with survival [59,62]. Folate receptor beta positive (FRβ^+^) TAMs, known to play a role in angiogenesis, are also a poor prognostic indicator in the pancreatic TME [62]. Similarly, patients with higher levels of infiltrating TEMs have a lower rate of 5-year overall survival [59]. The macrophages in the PDAC TME can be derived from recruited MonoMacs or from a priori TRMs within the pancreas. In a study by Zhu, et al., macrophages in the PDAC TME were found to consist of both MonoMacs and TRMs. The TRMs had low antigen presenting capabilities and higher expression of genes for extracellular matrix deposition and remodeling, which may contribute to the dense fibrotic stroma characteristic of PDAC [39]. These findings suggest a need for better understanding of macrophage ontogeny, function, and unique interactions with other cells in the TME. Thus, throughout this review, we will focus on how macrophages may interact with neutrophils in PDAC.

## 5. Introduction to Neutrophils in PDAC

Neutrophils are another highly important innate immune cell type infiltrating the PDAC TME. The proportion of neutrophils infiltrating the pancreatic tumor is increased compared to both healthy pancreas and chronic pancreatitis [5]. Furthermore, the neutrophil to lymphocyte ratio and the systemic inflammatory index, calculated by multiplying neutrophil and platelet counts and dividing by lymphocyte count, is a negative predictor of overall survival in PDAC [63,64,65]. Neutrophils have been identified as positive for CD66b, CD177, and CD15 [5,66] in humans or Gr-1 and Ly6G [67] in murine models. Several studies have shown that C-X-C motif chemokine ligands CXCL1-3, CXCL5-6, and CXCL8 bind to C-X-C motif chemokine receptors CXCR1 and CXCR2 expressed on neutrophils and play a role in recruitment into the TME [67,68,69,70]. Specifically, PDAC tumor cells can release chemokines such as CXCL1, granulocyte/macrophage colony-stimulating factor (GM-CSF), granulocyte colony-stimulating factor (G-CSF), and CXCL16 to induce neutrophil migration [71,72]. IL-17 [4] and transforming growth factor beta (TGFβ) [73] have also been shown to recruit neutrophils. Migration of neutrophils may also occur through ANG1 and ANG2 binding to the TIE2 receptor and signaling via the PI3k pathway [74]. The TIE2 receptor has been found on the cell surface of human neutrophils and aids in adhesion of human neutrophils to endothelial cells via increased production of platelet activating factor (PAF) and subsequent increase in β2-integrin [75]. ANG1 can also bind at the TIE2 receptor and stimulate release of IL-8, which in turn promotes neutrophil viability [76]. 

Studies of neutrophils have primarily focused on these cells as a homogenous cell population. Patients with high neutrophil infiltration have a worse prognosis in PDAC [5,66], which may be explained by studies indicating that tumor associated neutrophils (TANs) have a pro-tumorigenic phenotype in different cancer models [37,77,78,79,80]. In general, neutrophils infiltrating the TME at early stages may have anti-tumorigenic functions [37,81]. Similarly, for PDAC, neutrophils in the pancreas at the PanIN stage have greater migratory capacity without immunosuppressive qualities, but at later stages of disease, the neutrophils are less migratory and more immunosuppressive by suppression of T cell proliferation [37]. This demonstrates a need for greater understanding of the heterogenous nature of neutrophils in the TME. Importantly, differentiation between immunosuppressive PMN-MDSCs and immunosuppressive subtypes of neutrophils has not been well established [82]. Additionally, Evrad, et al. have identified a precursor neutrophil population (Lin^−^Gr1^+^CD11b^+^cKit^+^CXCR4^+^), an immature neutrophil population (Ly6G^lo/+^CXCR2^−^CD101^−^), and a mature neutrophil population (Ly6G^+^CXCR2^+^CD101^+^) with functional differences, highlighting the importance of understanding neutrophil heterogeneity. Immature neutrophils infiltrate the PDAC TME at a greater rate than the healthy pancreas. Further, tumor burden positively correlates with greater number of immature neutrophils in the blood and pancreas [83]. Neutrophils have also been suggested to assume different functions based on their activation status to an immunostimulatory (N1-like) or immunosuppressive (N2-like) subtype similar to the nomenclature used for macrophages. While N1-like and N2-like neutrophils have not been extensively characterized, N1-like neutrophils have hypersegmented nuclei compared to N2-like neutrophils [84]. Cell surface markers may also be used to determine N1-like and N2-like neutrophils. Fas receptor (FasR) and Intracellular Adhesion Molecule (ICAM)-1 are significantly elevated on neutrophils treated with immunostimulatory cytokines to polarize toward an N1-like state while CXCR2 is significantly elevated on N2-like neutrophils. Expression of CD62 ligand (CD62L) is another potential marker for neutrophil polarization. CD62L is known to be shed from the surface of the neutrophil once it is activated, and the cell surface expression of CD62L is lower on N1-like neutrophils than N2-like neutrophils. Similarly, release of myeloperoxidase (MPO), a catalyst for the formation of reactive oxygen species (ROS), is higher from N1-like neutrophils further indicating an activated state. In contrast, N2-like neutrophils secrete greater amounts of IL-8 than N1-like neutrophils, suggesting their capacity for recruiting additional neutrophils [85]. Regarding function, N1-like neutrophils produce T cell recruiting chemokines while N2-like neutrophils increase their production of ARG-1, which suppresses T cell function [84]. Both TGFβ [84] and G-CSF [86] can stimulate an immunosuppressive phenotype for neutrophils, but other factors contributing to neutrophil polarization are still under investigation. More work is needed to understand how neutrophils are polarized and function within the TME. 

In addition to polarization of neutrophils to immunostimulatory or immunosuppressive phenotypes, neutrophils have been studied for their ability to release extracellular traps of decondensed chromatin, DNA, and intracellular proteins or neutrophil extracellular traps (NETs) [87]. Originally, NETs were determined to have an antimicrobial function, but NETs have since been explored for their role in inflammatory diseases and cancer. All possible pathways leading to NETosis have not yet been elucidated, but several key enzymes have been identified. Peptidyl arginine deaminase 4 (PAD4) is important for NETosis and is responsible for citrullination of histones, which allows chromatin to unravel [88,89,90]. NADPH oxidase also contributes to NETosis through formation of ROS [91,92]; however, the role of NADPH oxidase appears to be dispensable as there are ROS independent NETosis pathways [90,93]. NETs can be identified in organs, such as the tumor, or in circulation, via markers of cell free (cf)-DNA, citrullinated histone 3 (CitH3), and MPO-DNA conjugates. The presence of NETs is a negative prognostic indicator for overall survival and recurrence free survival and has been suggested as an additional indicator for TNM (tumor size, local nodal involvement, and metastasis status) staging in PDAC. For patients receiving adjuvant chemotherapy, the presence of NETs is a negative predictor of overall survival [94]. Furthermore, postoperative NETs have been correlated with a decreased disease-free survival and an increased risk of metastases in colorectal cancer [95]. Studies have not only focused on how NETs contribute to PDAC progression but also how the TME might contribute to the formation of NETs or NETosis. Molecules such as IL-8, PAF, IL-17, and ANG1 and -2 induce NET formation [4,96]. Stimulation of the CXCR1 and CXCR2 receptors on neutrophils can stimulate NETosis [69]. Hypoxia, which is a significant component of the PDAC TME [97], may also play a role in the NETosis pathway. Downregulation of the transcription factor hypoxia inducible factor (HIF)-1α via an mTOR dependent pathway decreases NETosis [98] while increased NETosis occurs when HIF-1α is stabilized [99]. In general, when HIF-1α is stabilized under hypoxia, it forms a heterodimer with HIF-1β, which then translocates to the nucleus and binds to hypoxia response elements (HREs) to drive transcription of genes that regulate energy metabolism, apoptosis, blood vessel structure/function and angiogenesis with intent on oxygen recovery, and metabolic adaptation to hypoxic tissue situations. Importantly, the PAD4 promoter contains HREs, which explains increased NETosis under hypoxia [100]. It should also be noted that other studies have shown NETosis decreases under hypoxic conditions. Specifically, cholesterol accumulates in neutrophils under hypoxia, leading to decreased NETosis. Although the full mechanism has not been elucidated, the change in NETosis may be due to inhibition of NADPH oxidase-independent mechanisms of NETosis, which would be important when oxygen levels are low [101,102]. Despite conflicting literature regarding the role of hypoxia in NETosis, it is evident that PDAC contributes to NET formation as neutrophils derived from circulation have increased propensity to form NETs [103]. Given their diverse functions described here, it is likely that NETs and neutrophils affect the function of other cell types in the PDAC.

## 6. Spatial Arrangement of Macrophages and Neutrophils in the PDAC TME

While the presence of neutrophils positively correlates with CD68^+^ macrophages and CD163^+^ or CD204^+^ TAMs in the PDAC tumor [5], suggesting a relationship between these cell types, no studies have specifically focused on their spatial relationship or neutrophil-macrophage subset interactions during PDAC progression. Here, we discuss what is known in the literature and the shortcomings of our understanding of macrophage/neutrophil relationships in the PDAC TME. We further hypothesize how macrophages and neutrophils may be spatially oriented, particularly in hypoxic tumor areas. 

In general, macrophages within the PDAC TME (identified by the pan-macrophage marker CD68) have a M2-like TAM polarization [5]. These CD163^+^ or CD204^+^ TAMs are localized in malignant areas of the pancreas [5], specifically around vasculature [59] and near the invasive regions of the tumor [104]. CD163^+^ TEMs also preferentially infiltrate the tumor perivascular area [59]. In contrast, CD68^+^ HLA-DR^+^ M1-like macrophages are found in areas of non-malignant inflammation [5]. It is also important to note that the location of macrophages within the PDAC TME is not static, indicating the need to compare premalignant lesions and tumors at different stages. Further, previous studies of macrophage location within the PDAC TME used macrophage polarization for identification. Future studies could aim to identify macrophages based on ontogeny, including MonoMacs, TEMs, and TRMs as we have suggested in Figure 1. Literature discussing the spatial location of neutrophils in the PDAC TME is similarly limited. Neutrophil infiltration, like TAM infiltration, is greater in areas of malignancy when compared to areas of nonmalignant inflammation [5]. The neutrophils are primarily located near areas of densely packed neoplastic cells and decrease in prevalence moving away from these areas [105]. Importantly, previous studies of neutrophil infiltration do not identify differentially polarized neutrophils, maturity of neutrophils, or neutrophils having undergone NETosis; thus, identifying subclasses of neutrophils in these ways, as described above, may be important for understanding macrophage and neutrophil interactions within the PDAC TME. 

**Figure 2:**
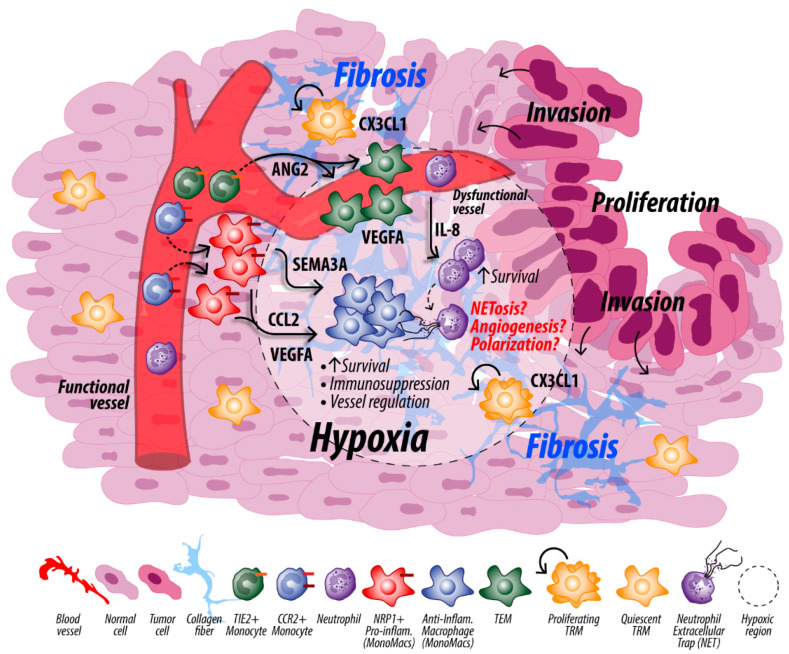
Hypothesized interactions between macrophages and neutrophils in areas of hypoxia. Both macrophages and neutrophils may be recruited to hypoxic regions within the tumor. Additional details about these interactions are discussed in the text.

Despite the lack of studies identifying macrophage/neutrophil spatial relationships in the PDAC TME, we hypothesize that regions of hypoxia represent one area where macrophages and neutrophils could be in close proximity and interact. When MonoMacs infiltrate the TME, they originally localize around the tumor vasculature through which they were recruited [106]. These macrophages can then be preferentially recruited to areas of hypoxia by CCL2 [107] or vascular endothelial growth factors (VEGF-A) [108]. The Sema3A/Nrp1 axis is also important for recruiting TAMs into areas of hypoxia. *Sema3A* is upregulated by hypoxic cancer cells to recruit TAMs, but TAMs then lose expression of the Sema3A receptor, *Nrp1*, and can become trapped [106]. MonoMacs recruited to areas of hypoxia may have improved survival, which has previously been demonstrated in vitro [109]. TEMs may also be recruited to areas of hypoxia via TIE2/ANG2 signaling and contribute to hypoxia [51]. Further, quiescent pancreatic TRMs that were recruited and maintained a priori may also be eventually engaged by tumor cell CX3CL1 overexpression [110] to proliferate and initiate persistent tissue remodeling and contribute to fibrosis via fibrogenesis and fibrinolysis [111]. Similarly, hypoxia increases neutrophil adhesion to endothelial cells and transmigration in a model of fibrotic interstitial lung disease, suggesting neutrophils are also recruited to areas of hypoxia [99]. Neutrophil survival is also enhanced under in vitro hypoxic conditions [112]. These findings suggest that macrophages and neutrophils are likely to be in close proximity in hypoxic regions of the PDAC TME (Figure 2). Further studies of the macrophage/neutrophil spatial relationship are warranted to better understand their crosstalk in PDAC.

## 7. Macrophage and Neutrophil Crosstalk in the PDAC TME

Thus far, studies of macrophage and neutrophil interactions have been primarily limited to macrophage efferocytosis, or phagocytic removal of apoptotic neutrophils to resolve inflammation, but such studies have not been conducted in cancer models. For example, neutrophils have been shown to undergo apoptosis [113] or NETosis [4] in the presence of IL-17 while macrophages are stimulated to efferocytose apoptotic neutrophils in the presence of IL-17 [113]. The ability of macrophages to remove apoptotic neutrophils may be decreased by NETs. Specifically, elastase released in NETs cleaves integrin α_v_β_3_ (a receptor for phagocytosis) from the surface of macrophages preventing efferocytosis of apoptotic cells in a model of sepsis [114]. In another study, TRMs, identified by expression of CD169, were shown to be responsible for “cloaking” sterile microlesions (necrosis of a few cells) to prevent neutrophil swarming from occurring at the lesion site. If these TRMs fail to cloak the microlesion and a neutrophil swarm occurs, these neutrophils are responsible for recruiting CCR2^+^ and CX3CR1^+^ MonoMacs, which then resolve the inflammation by removing neutrophil debris [115]. Given that TRMs and MonoMacs play different roles in recovery of inflammatory lesions in several non-cancer models, we hypothesize that these macrophage subtypes have different relationships with neutrophils and NETs in the PDAC TME. 

**Figure 3:**
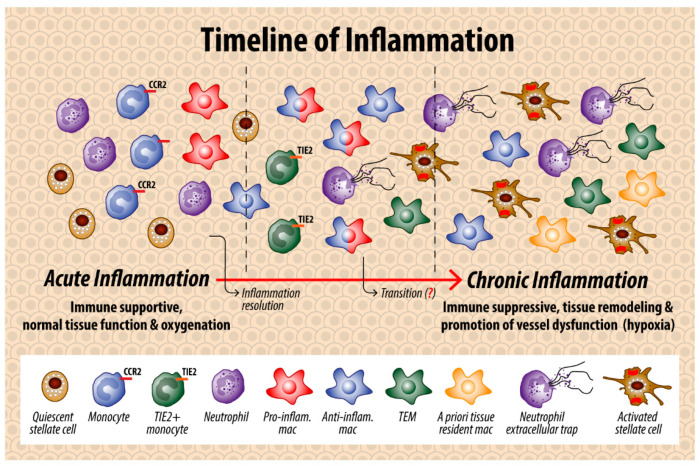
The timeline of inflammation during development of PDAC. Acute phases of inflammation can be resolved, but the inflammation fails to be resolved in the PDAC environment. This chronic, unresolving inflammation leads to immunosuppression, tissue remodeling, and vessel dysfunction.

Due to their interactions in various inflammatory models, macrophage and neutrophil crosstalk may be particularly important for understanding the progression of chronic pancreatitis, a risk factor for pancreatic cancer, to PDAC. In murine pancreatic tissue with acute pancreatitis, levels of CCL2 and CCR2 are elevated, suggesting MonoMac recruitment. In this cerulean-induced model, recruitment of MonoMacs may be detrimental since disruption of the CCR2/CCL2 axis decreases pancreatic damage. Interestingly, with disruption of the CCR2/CCL2 axis, there is a decrease in neutrophil recruitment in addition to the expected decrease in MonoMacs, suggesting that the interplay between these cells may cause tissue damage during bouts of pancreatitis [116]. In chronic pancreatitis, CCR2^+^ MonoMacs significantly contribute to macrophages within the pancreas. M2-like macrophages, with increased expression of CD206 and similar functions to TAMs as described in this review, predominate in this model of chronic pancreatitis [117]. M2-like macrophages have more anti-inflammatory processes and should contribute to resolution of inflammation and prevent tissue damage [118]. We hypothesize, however, that the presence of these M2-like macrophages in chronic pancreatitis generates a permissive environment for tumor development (Figure 3). 

**Figure 4:**
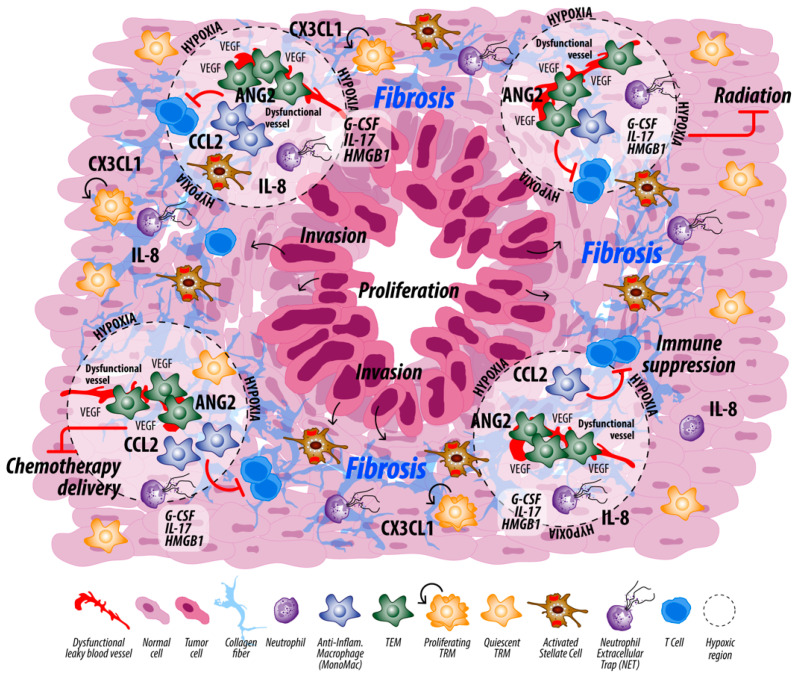
Recruitment of MonoMacs, TEMs, and neutrophils and proliferation of TRMs causes the unique TME in PDAC. MonoMacs, TEMs, and neutrophils are recruited via CCL2, ANG2, and IL-8, respectively. CX3CL1 causes recruitment and proliferation of TRMs. Together, these cells cause hypoxia, vascular remodeling, fibrosis, tumor progression, metastases, and immunosuppression. The sequalae of macrophage and neutrophil recruitment is further described in the text.

While the crosstalk between macrophages and neutrophils is important to preventing tissue damage from continuous inflammation in physiologic settings [119], it is likely more nuanced in the chronic inflammatory state of cancer (Figure 4). During the progression of PDAC in a pre-clinical model, both granulocytes and CD16/32^+^CD206^+^ TAMs increase in the pancreas [120]. In such a chronic inflammatory environment, neutrophils can release MPO. MPO in turn binds macrophage mannose receptor (CD206) to stimulate release of inflammatory cytokines/chemokines and ROS. The release of cytokines/chemokines results in recruitment of additional neutrophils to propagate the inflammatory response [121]. In later stages of the PDAC TME, neutrophils may assume a pro-tumorigenic phenotype [37,71], which is in part stimulated by presence of TGFβ [79,84,122,123]. Pancreatic cancer cells can also stimulate TAMs to release IL-8 and CCL2, which can recruit neutrophils and MonoMacs, respectively [124]. Furthermore, IL-1β released by tumor cells promotes accumulation of CD206^+^ TAMs and neutrophils into the PDAC TME [125]. IL-1β is also known to promote NETosis in neutrophils [126]. Additionally, such pro-inflammatory cytokines can increase the time until neutrophils undergo apoptosis [119,127], possibly contributing to the presence of neutrophils and NETs in the PDAC TME. Another interaction of note occurs for TEMs and TIE2-expressing neutrophils as ANG2 binding to the TIE2 receptor on both cell types results in release of IL-8, which promotes inflammation through recruitment and survival of neutrophils [76,128]. Given the presence of both macrophages and neutrophils in the PDAC TME, we hypothesize that macrophages fail to appropriately resolve inflammation, resulting in an ongoing immunosuppressive TME. Further, the dysregulated macrophage and neutrophil interactions promote a dysfunctional PDAC TME. 

## 8. Sequelae of Macrophages and Neutrophils in PDAC

### 8.1. Generation of Hypoxia 

Normal tissues maintain a delicate balance of angiogenic factor expression to preserve efficient blood vessel architecture and adequate delivery of oxygen, nutrients, and waste exchange in tissues during normal physiological processes, such as development or wound healing. VEGF, originally named vascular permeability factor, is a key driver of endothelial cell survival and proliferation required for angiogenesis [129]. In an acute setting of physiological hypoxia, VEGF production and angiogenesis are tightly regulated. Normal angiogenesis maintains an organized vessel tree comprised of functional vasculature that can perfuse blood into and throughout tissues and maintain normoxia, or normal oxygen levels for that specific tissue. However, the rapid proliferation of tumor cells surpasses the tissue diffusion distance of oxygen resulting in episodes of local, pathologic tumor hypoxia, in which the physiological demand for oxygen outweighs the supply [130]. Physiological hypoxia, experienced periodically by most healthy tissues, has been classified as 2% oxygen (15 mmHg) while pathological hypoxia resulting in disruption of tissue oxygen homeostasis is near 1% oxygen (or 8 mmHg). In the setting of pancreatic cancer, median oxygen levels have been measured as below 0.7% (0–5.3 mmHg) in comparison to adjacent tissues ranging from 1.2–12.3% (9.3–92.7 mmHg) [97,131]. Tumor hypoxia may be maintained or exacerbated by abnormal vasculature as PDAC is notorious for hypovascularity and poor perfusion [29]. It is important to note that even the vessels that do form in the PDAC TME may not function as vessels do in normal tissue. While normal vessels have strong pericyte coverage with tight junctions between endothelium to ensure stable, functional delivery of blood and oxygen, pathologic tumor hypoxia drives an imbalance between angiogenic factors (elevated ANG2:ANG1 ratio), resulting in dysregulated and non-productive angiogenesis. The resulting vasculature is tortuous and leaky with high vessel density but poor perfusion of blood and oxygen [132,133]. In human PDAC, hypoxia-stabilized HIF-1α expression in tumors correlated with expression of CCL2 and CD68, indicators of monocyte recruitment and macrophage presence, respectively [107]. In vitro analysis has also shown that HIF-1α regulates the transcription of CCL2 in PDAC cell lines and promotes recruitment of macrophages while PDAC-derived anti-inflammatory IL-35 indirectly recruits monocytes to the TME by a CCL5-dependent mechanism [134].

As previously discussed in this review (Figure 2), TEMs play a role in angiogenesis; however, in the setting of cancer, where the number of TEMs is elevated, TEMs are responsible for aberrant and dysfunctional angiogenesis [53]. In the transgenic rat insulin promoter (RIP)-T antigen (Tag) murine model of metastatic pancreatic neuroendocrine tumor (PNET), which causes carcinogenesis of β-cells of the pancreas and is commonly used to study tumor angiogenesis [135], inhibition of TIE2 on TEMs reduces TIE2^+^ myeloid infiltration resulting in reduced microvessel density, less vascular permeability, and blockade of tumor cell intravasation mediated by TIE2^Hi^/VEGF-A^Hi^ macrophages [136]. Importantly, these TEM numbers are reported to be regulated by hypoxia via HIF-1α-specific stabilization while the presence of HIF-2α in the macrophages appears to suppress HIF-1α-driven expansion. We recently reported that in a PyMT mouse model of breast cancer, breast tumors containing wild type and HIF-2α-deficient macrophages (containing only HIF-1α) had elevated numbers of tumor TEMs, increased hypoxia, and poor vessel perfusion that limited docetaxel delivery and efficacy. Mice with macrophages containing only HIF-2α (HIF-1α-deficient) had a reduced number of TEMs, better vascular architecture, and increased oxygen that enabled better perfusion and enhanced tumor cytotoxicity [53]. Like TEMs, a subset of neutrophils has been shown to express TIE2 [75,76]. Given that TEMs are associated with dysfunctional angiogenesis, TIE2-expressing neutrophils could similarly be explored for how hypoxia affects their recruitment and function in the PDAC TME and in turn how these neutrophils contribute to hypoxia. Further, to better understand how macrophages and neutrophils may generate hypoxia in the PDAC TME, it is important to note how these cells affect other factors contributing to hypoxia, such as vascular remodeling and fibrosis.

### 8.2. Vascular Remodeling

Vascular remodeling occurs during inflammation. In particular, capillaries are remodeled to venules, increasing permeability thus allowing leukocyte recruitment into the inflamed tissue [137]. Since neutrophils are among the first leukocytes recruited into inflamed tissue, they may serve as primary drivers of vascular remodeling and are known to secrete cytokines, growth factors, proteases, leukotrienes, histamine, and ROS, all of which critically affect vascular architecture [138,139,140,141]. Though inhibition of neutrophil infiltration promotes a more immature vessel structure [142] and neutrophil depletion alone inhibits cytokine-induced angiogenesis [143], there are few studies investigating the role of TANs in vascular remodeling within pancreatic tissue specifically. By investigating TAN function in other solid tumor models, we may begin to understand what role they may have in the PDAC TME. In a heterotopic Lewis lung carcinoma model, an increase in neutrophilic chemokine receptor CXCR2 function correlates with higher release of matrix metalloproteases, MMP-2 and MMP-9, as well as increased vessel density [144]. Further, Gr-1^+^ PMN-MDSCs coordinate the initial angiogenic switch in a RIP-Tag model of insulinoma by producing MMP-9, which increases the bioavailability of VEGF-A for its receptors, VEGFR-1 and -2, on endothelium [145]. Another group demonstrated that they could attenuate this effect by inducing placental growth factor-1 (PIGF-1) in the pancreatic islets, which decreased neutrophil recruitment [146]. In a murine syngeneic model of colon cancer, Gr-1^+^ myeloid cell recruitment correlates with vascular density [147]. Further, recruited neutrophils can contribute to vascular remodeling via direct binding to endothelial cells [148,149,150]. For example, murine knockout of leukocyte integrin α_M_β_2_ impairs vessel density and function in murine melanoma and prostate tumors. The neovessels in these knockout mice are leaky with fewer smooth muscle cells, lower pericyte coverage, reduced vessel diameter, and decreased basement membrane thickness indicating vessel immaturity [150]. The majority of the work presented here has been performed in tumor models other than pancreatic cancer, thus highlighting a need to elucidate the role of TANs on vascular remodeling in the PDAC TME. 

Much more work has been done on the contributions of macrophages in vascular remodeling in the pancreas when compared to neutrophils. The importance of macrophages in vascular remodeling is further demonstrated as TAM depletion decreases vessel density in the tumor of several pancreatic cancer models [151,152]. This effect may be initially coordinated by the tumor cells. Pancreatic cancer cells secrete high levels of adrenomedullin (ADM) that binds myelomonocytic cells to enhance their migration, invasion, and activity of MMP-2 in vitro. ADM knockdown or antagonism inhibits myelomonocytic cell recruitment, reduces vessel density, and suppresses PDAC progression [153]. In addition, conditioned media from cultured PDAC cells promotes TAM VEGF-induced HUVEC branching [154]. Radiotherapy may also affect the vascular remodeling capacity of macrophages. In a RIP-Tag transgenic model of PNET, low dose radiation-primed macrophages reduce VEGF-induced angiogenic proteins in HUVEC cells in vitro. Importantly, hypoxia-stabilized HIF-1α is down-modulated in these low dose radiation-primed macrophages resulting in polarization toward a M1-like phenotype and away from vessel dysfunction promoting M2-like TAMs [155]. This observation may be further explained by studies reporting that GM-CSF signals through HIF-2α to re-educate human and murine macrophages to become more M1-like while also stimulating their release of the anti-angiogenic molecule sVEGFR-1, an alternatively spliced isoform of membrane-bound VEGFR-1 to sequester excessive tumor VEGF leading to vessel regulation. For example, in alignment with the anti-angiogenesis field in the mid-2000s, we reported the vessel remodeling ability of intratumor injections of GM-CSF resulting in sequestration of tumor VEGF through TAM-derived sVEGFR-1 leading to increased hypoxia [156,157,158,159,160]. In recent works, we are testing intratumor delivery of GM-CSF using systemic PLGA/PEG-PLGA nanoparticles [161] as a method to remove excessive tumor VEGF that promotes vessel leakiness and aberrant sprouting, which in turn perpetuate tumor hypoxia leading to increased treatment resistance. While macrophages have been more thoroughly explored for their role in vascular remodeling in pancreatic cancer, additional studies are needed to understand how macrophages interact with other cells in the TME to influence this phenomenon.

In addition to independent functions, macrophages and neutrophils may coordinate to affect vascular remodeling in tumors. For example, Bv8, a mitogen for endothelium, regulates myeloid cell mobilization and is a neutrophil-derived mediator of tumor angiogenesis in several xenograft models [78,162]. CD11b^+^Gr1^+^ myeloid cells, including neutrophils, monocytes, and MDSCs, secrete Bv8 which acts directly on endothelial cells, enhancing angiogenesis in a colon carcinoma xenograft model. This effect has also been shown in a RIP-Tag transgenic murine model of PNET in which anti-Bv8 treatment inhibits CD11b^+^Gr1^+^ homing to neoplastic lesions resulting in a reduction in vascular surface area. Together, these data support the possibility of macrophage and neutrophil contribution of Bv8 in other solid tumors, such as PDAC [78]. In another study investigating polypeptide chemokine PK2 (Bv8, PROK2), antagonism with a small molecule inhibitor of PK2 reduced the F4/80^+^ TAM population in a PDAC xenograft model and improved response to chemotherapy [163]. However, the authors note that Bv8 inhibition did not change blood vessel density in the pancreatic cancer xenograft, attributing it to the hypovascular phenotype seen in human pancreatic cancer. Since vessel integrity was not investigated here, it is possible that Bv8 inhibition may modulate vessel function in these tumors, which enhanced the effect of chemotherapy. For example, we have shown that HIF-1α knockdown in myeloid cells decreases vessel density in a murine breast tumor model, but these vessels are more functional, with higher tumor oxygen tension, enhanced vessel perfusion, and ultimately a significant response to chemotherapy when compared to the wild-type controls [53]. It is also possible that macrophages recruit a specific pro-angiogenic subset of neutrophils to the TME through the VEGFR/VEGF-A axis. In particular, CXCR4-expressing neutrophils have been identified as a highly pro-angiogenic subtype of neutrophils that are recruited by VEGFR1/VEGF-A axis and produce MMP-9 to modulate revascularization via extracellular matrix digestion and release of matrix-bound growth factors in transplanted pancreatic islets [149,164]. Importantly, neutrophils do not express tissue inhibitors of matrix metalloproteinases (TIMPs), and therefore release TIMP-free MMP-9 [165]. In a syngeneic orthotopic murine PDAC model, TANs, not TAMs, predominantly produce MMP-9 in the metastatic environment, but proangiogenic VEGF expression colocalized with TAMs as opposed to TANs. Further, CD206^+^ CD163^+^ TAMs are found in murine PDAC metastases and produce VEGF and oncostatin M (OSM), a potent chemoattractant for neutrophils. TANs in this model produced MMP-9 [166]. As suggested above, MMP-9 from TANs may increase the bioavailability of VEGF-A for VEGFR2 on endothelium in this model, thus working together with TAMs to remodel the tumor vasculature. Therefore, it may be the coordinated effort of TAMs and TANs that contribute to the remodeling of the vasculature in the PDAC TME. 

### 8.3. Fibrosis

A dense, fibrotic stroma is a hallmark of PDAC that drives its pathogenesis by promoting hypoxia, limiting delivery of cytotoxic chemotherapy, and preventing immune cell infiltration. The dense stroma together with inadequate vessel structure serve as a barrier for oxygen diffusion through the tumor. The dense stroma also generates high tumor interstitial fluid pressure (IFP). High IFP in turn can prevent perfusion of existing blood vessels by leading to their collapse. In KPC GEMMs, the IFP of the tumor is almost 10 times higher than the normal pancreas. While the normal pancreas contains high amounts of blood vessels greater than 10 mm in diameter, KPC GEMM tumors contain no blood vessels above 10 mm in diameter, suggesting an important relationship between collapsed tumor blood vessels and high IFP [167], which may affect perfusion. This phenomenon has also been noted in subcutaneous xenograft models of PDAC [168] but should be further explored in human PDAC tumors.

Neutrophils and NETs have been implicated in the fibrosis that contributes to the pathophysiology of numerous disease processes [169]. NETs are known to activate lung fibroblasts [170] and have been associated with idiopathic pulmonary fibrosis, an irreversible chronic scarring in sporadic regions of the lungs, rich in collagen deposition [171]. PAD4^−/−^ mice, deficient in NETs, are protected from age-related cardiac and pulmonary fibrosis [172]. NETs have even been found in fibrotic tissue around orthopedic implants and associated with integration failure [173]. In PDAC, there are several mechanisms through which neutrophils promote pancreatic fibrosis [174]. Obesity induced recruitment of TANs results in pancreatic stellate cell activation, promoting desmoplasia [175]. NETs also activate pancreatic stellate cells, influencing pancreatic tumor microarchitecture [176]. PAD4^−/−^ mice have diminished α-SMA, a marker of stellate cell activation, in pancreatic tumors. DNA released from neutrophils during NETosis is responsible for increased α-SMA expression in pancreatic stellate cells and promotes their proliferation in vitro [87]. NET activation of fibroblasts is not limited to the pancreatic TME, as NETs enhance migration of hepatic stellate cells to liver micro-metastases and result in accumulation of cancer-associated fibroblasts in metastatic pancreatic tumors [177]. Overall, the importance of neutrophils/NETs in fibrosis, particularly in PDAC, has been well studied and can be linked to the hypoxia seen in the PDAC TME. 

The role of TAMs in the fibrotic TME of PDAC has also been investigated. In chronic pancreatitis, which increases risk for PDAC, macrophages facilitate fibrosis by producing TGFβ and platelet-derived growth factor (PDGF)-β [178]. As a source of IL-4/IL-13, pancreatic stellate cells promote M2-like macrophages (derived from both MonoMacs and TRMs) in a murine model of chronic pancreatitis. Meanwhile, blockade of IL-4/IL-13 in this model decreases M2-like macrophages and pancreatic fibrosis [117]. In the PDAC TME, MonoMacs may be recruited via CCL2 released from tumor cells. In an in vitro system, macrophages derived from circulating monocytes can activate pancreatic stellate cells isolated from primary pancreatic paraneoplastic tissue, increasing their expression of α-SMA [107]. Further, significant portions of TRMs expand during murine PDAC progression and exhibit a pro-fibrotic transcriptional profile and a strong trend towards poor survival when mapped to human PDAC datasets. This profile highlights molecules that modulate the extracellular matrix, such as ECM molecules (collagen isoforms, tenascin C, elastin), ECM-producing enzymes (hyaluronan synthases 2 and 3), and ECM-remodeling molecules (lysyl oxidase). Embryonically derived TRMs can produce collagen I and IV ex vivo, and depletion of these macrophages in vivo reduces collagen density in PDAC. Together, these data underscore a critical role for embryonically derived TRMs in PDAC fibrosis. In addition, CXCR4 is almost exclusively upregulated in embryonically derived TRMs from the murine PDAC model, and up to 40% of TAMs in human PDAC tissue expressed high levels of CXCR4 [39]. Given that pro-angiogenic TEMs also express high levels of CXCR4, we hypothesize that TEMs may serve as a subset of the pro-fibrotic resident macrophage [179,180]. We speculate that TEMs lose their angiogenic function and are converted to a pro-fibrotic phenotype in the PDAC TME, contributing to its hypovascularity. Overall, the crosstalk among neutrophils, macrophages, and pancreatic stellate cells discussed here may be a key mediator of macrophage-induced fibrosis in PDAC.

### 8.4. Immunosuppression

Both neutrophils and macrophages contribute to the immunosuppressive microenvironment characteristic of PDAC. Across several cancer types, including pancreatic cancer, TAMs are found to be a predictor of adverse outcomes [20]. TAMs may upregulate VCAM-1 in pancreatic cancer cells via secretion of CCL18, resulting in increased migration, invasion, and survival. VCAM-1 expression in pancreatic cancer cells also promotes Warburg metabolism, and the lactate released in turn stimulates macrophages to a more immunosuppressive TAM phenotype higher in *Cd206*, *Cd163*, and *IL-10* [181]. Regarding specific inhibitory functions of macrophages, Dectin-1 expression can cause immunosuppressive effects by inhibiting T cell function through the Dectin-1/galectin-9 pathway. Dectin signaling stimulates release of IL-10 from CD4^+^ T cells and inhibits CD8^+^ T cell activation [182]. IL-10 can also be released by TAMs and suppress release of IL-12 from TAMs [183] and dendritic cells [184], which in turn inhibits CD8^+^ T cell activation. TAMs in the PDAC TME can also express programmed death ligand 1 (PD-L1), which causes T cell dysfunction and decreases markers of T cell activation, such as interferon (IFN)-γ, Granzyme B, and perforin [185]. Demonstrating the importance of PDAC TAMs on T cell function, antagonism of CCR2 prevents CCR2^+^ MonoMacs from infiltrating the PDAC TME and also causes an increase both CD4^+^ and CD8^+^ tumor infiltrating lymphocytes [186]. 

More recently, neutrophils have been identified as having immunosuppressive capabilities. Tumor neutrophils may achieve a more immunosuppressive state via transition to oxidative metabolism [187,188]. Tumor cells release stem cell factor, stimulating c-Kit receptors on neutrophils and allowing neutrophils to utilize mitochondrial metabolism to produce ROS. The released ROS can then suppress nearby CD4^+^ T cells [187]. Specifically, in a metastatic model of PDAC, neutrophils expressing the purinergic receptor P2RX1 have N1-like markers while those lacking P2RX1 are positive for N2-like markers, including checkpoint protein PD-L1 [188]. Neutrophils having achieved a N2-like phenotype can suppress the activity of CD8^+^ T cells in the TME. One method is by upregulation of *Arg1*, and the ARG-1 protein uses up L-arginine critical for CD8^+^ T cell function [189]. Neutrophils found in later stages of PDAC can also suppress proliferation of T cells [37]. Another granulocytic cell type similar to immunosuppressive neutrophils is the PMN-MDSC. Elevated PMN-MDSCs have been identified in PDAC patients. Expression of high levels of CD13 on the surface of these PMN-MDSCs is associated with increased expression of ARG-1 and T cell suppression [190]. Further, PMN-MDSCs, through HIF-1α signaling, can upregulate PD-L1 [191]. In an acute setting, stabilization of HIF-2α can also cause *Arg1* upregulation [192]. In addition to causing immunosuppression through their own functions, neutrophils in the PDAC TME can also recruit other immunosuppressive cell types. For example, TANs can promote recruitment of immunosuppressive Tregs and MonoMacs to the TME through release of chemokines CCL17 and CCL2, respectively [79,193]. In addition to neutrophils, NETs have been explored for how they can influence immune cell function. For example, NETs can exclude CD8^+^ T cells from the TME serving as a physical barrier to T cell infiltration [3,4] and can specifically block NK cell or CD8^+^ T cell mediated cytotoxicity of tumor cells [69]. Although less commonly explored for their immunosuppressive qualities compared to macrophages, neutrophils/NETs in the TME can be detrimental to an anti-tumorigenic immune response. The immunosuppression caused by both macrophages and neutrophils are just one mechanism by which they contribute to tumor growth and metastasis. 

### 8.5. Tumor Progression and Metastasis 

In addition to causing immunosuppression, macrophages and neutrophils can directly affect the growth and metastasis of a tumor. As previously discussed, patients with increased infiltration of TAMs at the primary tumor site have a worse prognosis [59,62,104]. Further, implantation of tumor cells after co-culture with a human macrophage cell line results in greater tumorigenesis and metastasis than tumor cells alone in a pancreatic carcinoma orthotopic xenograft model [194]. Myeloid cells, including TAMs and M-MDSCs, can release IL-6 and stimulate signal transducer and activator of transcription (STAT)-3 activation and PDAC development [195,196]. TAMs from in vitro cell models or tumor tissues co-cultured with PDAC cells can also promote epithelial–mesenchymal transition (EMT) [197,198]. Similarly, TAMs are associated with increased activity of MMP-2 and MMP-9 from PDAC cells and a decrease in E-cadherin expression [198], which may promote the ability of tumor cells to metastasize. IL-8, which can also be released by TAMs, promotes PDAC tumor cell invasiveness [124,199]. TAMs have also been found to assist in promoting metastasis within PDAC tissue through the release of exosomal microRNA-501-3p (*miR-501-3p*), an inhibitor of the tumor suppressor TGFBR3, to activate the TGFβ signaling pathway and drive tumor cell migration [200]. Exosomes, also released by pancreatic tumor cells, can be taken up by Kupffer cells (macrophages in the liver) and promote a metastatic niche, including increased infiltration of MonoMacs and increased fibronectin production by pancreatic stellate cells [201]. Early recruitment of granulin secreting “metastasis-associated macrophages” or MAMs may also activate hepatic stellate cells to support metastatic growth [202]. Overall, macrophages can contribute to growth of the primary tumor and development of metastases through a number of different mechanisms. 

Tumor neutrophil infiltration has also been associated with metastatic potential in PDAC [203]. Neutrophils and NETs have been implicated in multiple steps in the pathway toward development of cancer metastases. Neutrophil elastase degrades E-cadherin and promotes EMT [204,205]. NETs can also sequester tumor cells within the circulation [206] and promote their adhesion in liver sinusoids via Mac-1 binding to ICAM-1 [207]. Further, neutrophil elastase, present in NETs, loosens endothelial cell junctions by degradation of VE-cadherin to promote vascular leakage [208] and enhance cancer cell motility [209] to drive EMT. NETs even cause an accumulation of cancer-associated fibroblasts in liver micro-metastases to support a pro-metastatic niche [177]. Proteases neutrophil elastase and MMP-9 are released from NETs and induce proliferation of dormant metastatic cancer cells, resulting in formation of metastatic tumors [210]. As a result of these numerous mechanisms, NETs have been associated with development of metastases in pancreatic cancer [211]. The presence of both neutrophils/NETs and macrophages in the PDAC TME plays a role in tumor progression and metastasis. Not only are these cells important for propagating tumor growth and metastasis but they may also play an important role in therapeutic resistance. 

### 8.6. Treatment Resistance 

Therapeutic resistance in PDAC is highly problematic and has commonly been attributed to the highly fibrotic and immunosuppressive nature of the PDAC TME. As previously discussed, NETs can stimulate pancreatic stellate cells causing fibrosis [87]. Not only do NETs stimulate fibrosis in the PDAC TME, but the NETs themselves may cause resistance to immunotherapies. A study by Teijeira, et al. showed that NETs surround tumor cells preventing contact by cytotoxic NK cells and CD8^+^ T cells [69]. Antibodies against IL-17, used to inhibit NETosis, improve response to immune checkpoint blockade [4]. 

Gemcitabine, one of the chemotherapy agents commonly used to treat PDAC patients, increases immunosuppressive TAMs in the TME [212]. TAMs in the PDAC TME have been suggested to promote therapeutic resistance to gemcitabine through release of deoxycytidine, for which gemcitabine is an analog [213]. TAMs may also promote resistance to gemcitabine through release of TGFβ1 [214]. A study by Zhang, et al. also suggested that the crosstalk between TAMs and tumor cells in PDAC blocks success of monoclonal antibody treatment by upregulating CD59 expression on tumor cells, which inhibits complement dependent cytotoxicity [215]. Given these findings, depleting F4/80^+^ TAMs from the PDAC tumor has been suggested to improve response to gemcitabine [216].

Elevated CCL2 has been found in PDAC patients; thus, targeting recruitment of monocytes via the CCR2/CCL2 axis has been explored [10,70,186]. Importantly, blocking CCR2-recruited MonoMacs via CCR2 antagonist treatment improves response to FOLFIRINOX (5-fluorouracil, oxaliplatin, irinotecan, leucovorin) therapy in PDAC [186]. In response to treatment with a CCR2 antagonist, which inhibits MonoMac infiltration into the PDAC TME, there is a corresponding increase in neutrophil infiltration into the tumor [70]. To describe this phenomenon, Pahler, et al. demonstrated that macrophages can release factors that inhibit CXCL8-mediated neutrophil recruitment into the TME [217]. Without such inhibition, neutrophils may be free to enter the tumor. These findings demonstrate the intrinsic relationship between macrophages and neutrophils. Thus, to overcome therapeutic resistance in PDAC, targeting both macrophages and neutrophils may be an important strategy to consider due to the intrinsic relationship between the two cell types.

## 9. Treatments Targeting Macrophages and Neutrophils 

Many methods have been used to target either macrophages or neutrophils in PDAC (Table 2). Re-educating TAMs has been suggested to be key in overcoming therapeutic resistance [120,212]. When TAMs are treated with IFNγ, they acquire characteristics of M1-like, immunosupportive macrophages, including increased release of pro-inflammatory IL-12 as well as decreased release of pro-tumorigenic cytokines IL-10, MMP-9, VEGF, and CCL18 [218]. In addition, inhibition of TAM MMP-9 expression decreased tumor growth and tumor neovasculature in pre-clinical models of cervical carcinoma [219]. TAMs also can be re-educated through binding of CD40 by a CD40 agonistic antibody. CD40 is present on antigen presenting cells, including monocytes, dendritic cells, and B cells, and binding of CD40 by an agonistic antibody or its ligand CD154 results in cell activation [220]. Specifically, F4/80^+^ TAMs in the PDAC TME express CD40, and treatment with a CD40 agonistic antibody causes upregulation of MHCII and co-stimulatory molecule CD86 and release pro-inflammatory cytokines [221]. A Phase 1b clinical trial with addition of a CD40 agonist to gemcitabine plus nab-paclitaxel showed improved clinical activity in PDAC [220]. In a pre-clinical model of metastatic PDAC, addition of a CD40 agonist improves response to T cell activation strategies, such as PD-1 blockade [222]. Addition of a CD40 agonist in combination with radiotherapy also improves response to dual immune checkpoint blockade [223]. In another method for reprogramming macrophages, inhibition of the PI3k-γ and CSF1-R pathways via a nanomicelle formulation targeted to macrophages causes reprogramming of M2-like TAMs to a M1-like phenotype. This treatment causes an increase in CD4^+^ and CD8^+^ T cell infiltration and decrease in CD45^+^ CD11b^+^ Gr-1^+^ MDSC infiltration [224]. Additionally, treatment with pomalidomide decreases immunosuppressive TAMs but not immunosupportive M1-like macrophages in PanIN lesions and the PDAC TME. The resulting TME shows decreased fibrosis and increased infiltration of activated CD4^+^ and CD8^+^ T cells [225].

In addition to reprogramming macrophages, their recruitment and survival may be targeted to deplete them from the TME. Importantly, pre-clinical studies have shown that lurbinectedin treatment to deplete F4/80^+^ TAMs from the PDAC tumor can improve response to gemcitabine [216]. When CSF1 or CCR2, both important for recruitment and maintenance of macrophages in the TME, are inhibited in combination with gemcitabine therapy, there is improved control of tumor growth in pre-clinical models. There is also an increase in infiltrating CD4^+^ and CD8^+^ T cells and a decrease in infiltrating Tregs [226]. When a CCR2 antagonist is added to FOLFIRINOX therapy for patients with PDAC, local control of the tumor can be achieved [186]. Further, combining an antibody against CCL2 with radiotherapy decreases tumor size and improves survival time in a murine model of PDAC [227]. Liposomal clodronate can also be used to deplete TAMs resulting in decreased tumor vessel density, tumor volume, and metastasis [152,228]. Overall, these studies suggest that removal of macrophages from the PDAC TME may also prove to be a useful strategy and improve therapeutic results. 

Given the prominence of neutrophils and NETs in PDAC, they have also been attractive targets for overcoming therapeutic resistance in PDAC. Due to the complex biology of neutrophil functions, some studies have suggested removing neutrophils from the TME altogether. For example, CXCR2 signaling at the tumor border is linked to worse outcomes for patients with PDAC. Since CXCR2 is an important receptor for recruitment of neutrophils, the addition of CXCR2 inhibition to gemcitabine treatment improves overall survival and improved sensitivity to anti-PD-1 treatment [229], which may be due to an increase in T cell infiltration [67]. Treatment with lorlatinib, a small molecule tyrosine kinase inhibitor, also suppresses neutrophil infiltration into the PDAC TME via inhibition of the non-receptor tyrosine kinase FES. Nielson, et al. suggest that lorlatinib decreases neutrophils in the PDAC TME by inhibiting development of neutrophils in the bone marrow and inhibiting their migration. Addition of lorlatinib to gemcitabine treatment has no additive effect; however, lorlatinib improves response to anti-PD-1 treatment in a pre-clinical model of PDAC [71]. 

Other strategies have focused on reprogramming neutrophils. IFNβ has been proposed to stimulate neutrophils toward a more N1-like or anti-tumorigenic phenotype [230]. In addition, blockade of TGFβ increases neutrophil production of immunostimulatory cytokines and cytotoxicity toward tumor cells [84]. Still, other methods have focused on targeting NETosis as NETs have been implicated in a worse prognosis for patients with PDAC [94]. In murine models of PDAC deficient in NETs, there is improved overall survival and decreased tumor volume. Dnase treatment has a similar effect in pre-clinical models [87]. Chloroquine has also been shown to inhibit NET formation in pre-clinical models of acute pancreatitis and PDAC [231,232]. In a Phase II clinical trial, adding hydroxychloroquine, the less toxic derivative of chloroquine, to gemcitabine and nab-paclitaxel treatment, the response rate was improved for patients with metastatic PDAC. The overall survival rate after 12 months did not improve, suggesting a need for a biomarker for PDAC patients receiving hydroxychloroquine therapy [233]. In other Phase I/II trials, however, patients receiving hydroxychloroquine had improved histopathological response. Further, patients with elevated levels of carbohydrate antigen (CA) 19-9, a protein marker commonly elevated in PDAC, who had a decrease to normal levels had improved overall and recurrence-free survival. This suggests that CA 19-9 levels can be used to monitor outcomes in patients receiving hydroxychloroquine therapy [103,234]. Other methods of targeting NETosis include blockade of cytokine signaling. For example, IL-17 stimulates NETosis and prevents infiltration of CD8^+^ T cells into the PDAC TME. Combined treatment using IL-17 blockade with dual checkpoint blockade improved control of tumor growth in an orthotopic pre-clinical model of PDAC [4]. More targeted methods focus on inhibition of PAD4, a critical enzyme in the formation of NETs. PAD4 inhibition decreases NET formation in both murine and human neutrophils [89]. 

To overcome therapeutic resistance in PDAC, combination therapies targeting TAMs, neutrophils, and/or NETs may be an important strategy to consider due to the intrinsic relationship between the two cell types described throughout this review. In response to treatment with a CCR2 antagonist, there is a corresponding increase in neutrophil infiltration into the PDAC TME. Nywening et al. thus suggested the addition of a CXCR2 inhibitor to improve the efficacy of CCR2 antagonist with FOLFIRINOX therapy. The combination of a CCR2 antagonist, CXCR2 inhibitor, and FOLFIRINOX improved survival outcomes in an orthotopic pre-clinical model of PDAC [70]. The chemotherapy agent trabectedin preferentially depletes monocytes and TAMs via caspase-8-specific activation without a significant change in neutrophil, T cell, and B cell populations. Importantly, a compensatory increase in neutrophils is not seen in treatment with trabectedin compared other methods of depleting TAMs [235], which may indicate trabectedin as a potential candidate for TAM depletion in PDAC. Nonetheless, the apparent link between macrophages and neutrophils in the PDAC TME will likely require that both cell types be targeted to overcome therapeutic resistance in PDAC. 

**Table 2:** Possible therapeutic strategies that can be used to target macrophages and neutrophils in PDAC.

Mechanism	Treatment	References
Macrophage Reprogramming	IFNγ	[218]
	MMP-9 Inhibitor	[219]
	CD40 agonistic antibody	[220,221,222,223]
	Nanomicelle PI3k-γ and CSF1-R Inhibition	[224]
	Pomalidomide	[225]
	Trabectedin	[235]
Macrophage Depletion	Lurbectedin	[216]
	CSF1 Inhibition	[226]
	CCR2 Antagonism	[70,186,226]
	CCL2 Antibody	[227]
	Liposomal Clodronate	[152,228]
Neutrophil Reprogramming	IFNβ	[230]
	TGFβ	[84]
Neutrophil Depletion	CXCR2 Inhibition	[70]
	Lorlatinib	[71]
NETosis Inhibition	Dnase	[87]
	(Hydroxy)Chloroquine	[103,231,232,233,234]
	IL-17/IL-17R Blockade	[4]
	PAD4 Inhibition	[89]

## 10. Conclusions

Given the expansive literature presented throughout this review, we hypothesize that neutrophils and several macrophage subtypes present in the PDAC TME cause an unresolving chronic inflammatory environment (Figure 4). The presence of elevated levels of MonoMacs, TEMs, TRMs, and neutrophils in the TME is not only unique to any normal tissues during injury or infection but also unique to PDAC compared to other solid tumor types (Figure 1). We hypothesize that, since resolution of inflammation requires a careful orchestration of neutrophil and macrophage recruitment and functions, there may be several steps at which this failure to resolve inflammation can occur. In acute inflammation, TRMs fail to cloak microlesions that occur at the inflammatory site [115]. As a result, neutrophils are recruited. Subsequently, MonoMacs are recruited into the environment to resolve the inflammation. These MonoMacs should be responsible for resolving the inflammation as occurs in acute pancreatitis. If MonoMacs fail to resolve the inflammatory stimulus, however, chronic inflammation can occur. In the pancreas, this may occur as chronic pancreatitis, which is a known risk factor for pancreatic cancer. We hypothesize that this increased risk of pancreatic cancer is due to the unresolving inflammatory insult orchestrated by a dysregulated neutrophil/macrophage axis. Importantly, even in the absence of clinical pancreatitis, chronic inflammation is a hallmark of pancreatic cancer. Rather than forming an adequate immune response to combat pancreatic cancer cells, the neutrophils and macrophages have aberrant functions that may perpetuate conditions contributing to tumor development. In particular, the recruited neutrophils may be immunosuppressive or form NETs. The NETs can contribute to the fibrotic nature of PDAC by activating pancreatic stellate cells. Due to the continuous inflammation that is not resolved by MonoMacs, tissue remodeling TRMs may also become activated and proliferate. TRMs have poor antigen presenting capabilities and pro-fibrotic characteristics [39], which contribute to both the immunosuppressive and fibrotic qualities of the PDAC tumor. Further, TEMs, which typically have pro-angiogenic function in well controlled tissues during development, can contribute to the dysfunctional and hypoxic TME. Given the hypovascular nature of the PDAC TME, we hypothesize that TEMs could fail to support adequate blood vessel function or drive vessel dysfunction. As a result of the aberrant functions of these TEMs, there can be a failure of delivery of therapeutic agents, successful implementation of radiotherapy, or infiltration cytotoxic T cells (Figure 4); however, the role of these TEMs and angiogenesis should be further explored in the context of PDAC since hypovascularity has been previously thought to limit metastasis. Our extensive literature search has led us to hypothesize that both macrophages and neutrophils are responsible for the uniquely fibrotic and immunosuppressive PDAC TME and cooperate to play crucial roles in PDAC progression and therapeutic resistance. Therefore, the interactions and functions of both macrophages and neutrophils within the PDAC TME require further exploration, and these studies may lead to the discovery of novel targets for PDAC immunotherapies. 

## Figures and Tables

**Table 1:** Characterization of macrophage subtypes by function and markers. This table does not include pan-macrophage markers, such as CD11b (Myeloid lineage marker), F4/80 (Murine), and CD68 (Human).

Macrophage Subtype	Function	Murine Markers	Human Markers
M1-like	Immunosupportive	CD80^+^ CD86^+^ MHCII^+^ iNOS^+^	CD80^+^ CD86^+^ HLA-DR^+^ iNOS^+^
M2-like/TAM	Anti-inflammatory, Immunosuppressive	CD206^+^ ARG1^+^ FRβ^+^	CD206^+^ CD204^+^ CD163^+^ ARG1^+^ FRβ^+^
TEM	Anti-inflammatory, Immunosuppressive, Angiogenesis	TIE2^+^ CD31^−^	TIE2^+^ CD31^−^
MonoMac	Pro-inflammatory, Immunosupportive	CCR2^+^ Ly6C^hi^ CX3CR1^lo^	CCR2^+^ CD14^++^ CD16^−^
TRM	Cloak microlesions, Tissue remodeling	CX3CR1^hi^ Ly6C^lo^ CCR2^−^	CX3CR1^+^

## Abbreviations

Pancreatic ductal adenocarcinoma, PDAC; tumor microenvironment, TME; tumor associated macrophage, TAM; tumor associated neutrophil, TAN; regulatory T cells, Tregs; myeloid derived suppressor cell, MDSC; polymorphonuclear myeloid derived suppressor cells, PMN-MDSC; monocyte-derived macrophage, MonoMac; endothelial tyrosine kinase receptor, TIE2; TIE2-expressing macrophage, TEM; tissue resident macrophage, TRM; genetically engineered mouse model, GEMM; pancreatic/duodenal homeobox protein-1, PDX-1; PTF1a-p48 promoter, p48; *Pdx-Cre*; *K-ras*^*LSL*.G12D/+^; *p53^R17H/+^*, KPC; *Pdx-Cre; K-ras^LSL^*^.G12D/+^, KC; pancreatic intraepithelial neoplasm, PanIN; patient derived xenograft, PDX; lipopolysaccharide, LPS; LPS-binding protein, LBP; toll-like receptor 4, TLR4; myeloid differentiation factor 2, MD2; interleukin, IL; Fc-gamma Receptor III, FcγRIII; C-C Motif Chemokine Receptor 2, CCR2; C-C Motif Chemokine Ligand 2, CCL2; Arginase-1, ARG-1; Angiopoietin ligand, ANG; Folate receptor beta, FRβ; granulocyte/macrophage colony-stimulating factor, GM-CSF; granulocyte colony-stimulating factor, G-CSF; transforming growth factor beta, TGFβ; platelet activating factor, PAF; Fas receptor, FasR; Immunosupportive macrophage, M1-like; Immunosupportive neutrophil, N1-like; Immunosuppressive macrophage, M2-like; Immunosuppressive neutrophil, N2-like; Intracellular Adhesion Molecule 1, ICAM-1; CD62 ligand, CD62L; myeloperoxidase, MPO; reactive oxygen species, ROS; neutrophil extracellular traps, NETs; Peptidyl arginine deaminase 4, PAD4; cell free DNA, cf-DNA; citrullinated histone 3, CitH3; hypoxia inducible factor, HIF; hypoxia response elements, HREs; vascular endothelial growth factors, VEGF-A; mannose receptor, CD206; rat insulin promoter-T antigen, RIP-Tag; pancreatic endocrine tumor, PNET; matrix metalloproteinase, MMP; placental growth factor-1, PIGF-1; polypeptide chemokine PK2, Bv8, PROK2; tissue inhibitors of matrix metalloproteinases, TIMPs; interstitial fluid pressure, IFP; platelet-derived growth factor-β, (PDGF)-β; programmed death ligand 1, PD-L1; interferon, IFN; signal transducer and activator of transcription, STAT; epithelial-mesenchymal transition, EMT; 5-fluorouracil oxaliplatin irinotecan leucovorin therapy, FOLFIRINOX; carbohydrate antigen 19-9, CA 19-9.
